# Plasticized Sodium-Ion Conducting PVA Based Polymer Electrolyte for Electrochemical Energy Storage—EEC Modeling, Transport Properties, and Charge-Discharge Characteristics

**DOI:** 10.3390/polym13050803

**Published:** 2021-03-05

**Authors:** Shujahadeen B. Aziz, Muaffaq M. Nofal, Rebar T. Abdulwahid, Hewa O. Ghareeb, Elham M. A. Dannoun, Ranjdar M. Abdullah, M. H. Hamsan, M. F. Z. Kadir

**Affiliations:** 1 Hameed Majid Advanced Polymeric Materials Research Lab., Physics Department, College of Science, University of Sulaimani, Qlyasan Street, Sulaimani 46001, Iraq; rebar.abdulwahid@univsul.edu.iq (R.T.A.); ranjdar.abdullah@univsul.edu.iq (R.M.A.); 2 Department of Civil Engineering, College of Engineering, Komar University of Science and Technology, Sulaimani 46001, Iraq; 3 Department of Mathematics and General Sciences, Prince Sultan University, P.O. Box 66833, Riyadh 11586, Saudi Arabia; muaffaqnofal@gmail.com; 4 Department of Physics, College of Education, University of Sulaimani, Old Campus, Sulaimani 46001, Iraq; 5 Chemistry Department, College of Science, University of Sulaimani, Qlyasan Street, Sulaimani 46001, Iraq; hewa.ghareeb@univsul.edu.iq; 6 Associate Director of General Science Department, Woman Campus, Prince Sultan University, P.O. Box 66833, Riyadh 11586, Saudi Arabia; elhamdannoun1977@gmail.com; 7 Centre for Foundation Studies in Science, University of Malaya, Kuala Lumpur 50603, Malaysia; hafizhamsan93@gmail.com (M.H.H.); mfzkadir@um.edu.my (M.F.Z.K.)

**Keywords:** PVA, NaI, glycerol, polymer electrolyte, impedance, ion transport parameters, EDLC device

## Abstract

This report presents the preparation of plasticized sodium ion-conducting polymer electrolytes based on polyvinyl alcohol (PVA)via solution cast technique. The prepared plasticized polymer electrolytes were utilized in the device fabrication of electrical double-layer capacitors (EDLCs). On an assembly EDLC system, cyclic voltammetry (CV), electrical impedance spectroscopy (EIS), linear sweep voltammetry (LSV), transfer number measurement (TNM) and charge–discharging responses were performed. The influence of plasticization on polymer electrolytes was investigated in terms of electrochemical properties applying EIS and TNM. The EIS was fitted with electrical equivalent circuit (EEC) models and ion transport parameters were estimated with the highest conductivity of 1.17 × 10^−3^ S cm^−1^ was recorded. The CV and charge-discharging responses were used to evaluate the capacitance and the equivalent series resistance (ESR), respectively. The ESR of the highest conductive sample was found to be 91.2 Ω at the first cycle, with the decomposition voltage of 2.12 V. The TNM measurement has shown the dominancy of ions with *t_ion_* = 0.982 for the highest conducting sample. The absence of redox peaks was proved via CV, indicating the charge storing process that comprised ion accumulation at the interfacial region. The fabricated EDLC device is stable for up to 400 cycles. At the first cycle, a high specific capacitance of 169 F/g, an energy density of 19 Wh/kg, and a power density of 600 W/kg were obtained.

## 1. Introduction

The modern lifestyle has required wide scale consumption of fossil fuels widely, causing massive environmental pollution. This has urged us to look for alternatives that are characterized by renewability and sustainability. Herein, several different types, such as wind, fuel cells, solar cells, geothermal and biofuels, have been considered to replace fossil fuels in industries [1,2]. The conventional organic sol-gel electrolytes have recently been replaced by new impressive electrolyte materials known as solid polymer electrolytes (SPEs). These electrolytes have many advantages such as being harmless, the ease of processability and chemical, electrochemical and dimensional stabilities [3,4].

The polar polymers being ionically conductive incorporated with additives, for instance, metal salts, are less well-studied in electrochemical devices, especially lithium batteries, compared to other electrochemical devices [5,6]. Polar polymers can dissolve both inorganic and transition metal salts since a strong interaction occurs between the unshared pair electron of the heteroatom; nitrogen or oxygen of the polymer on one side and cation of the dopant salt on the other side.

Natural polymers are candidates of choice in preparing solid biopolymer electrolytes [7,8,9,10,11]. Herein, polyvinyl alcohol (PVA), as one of the natural polymers, is benign and contains polar oxygen atoms from the vinyl alcohol groups. This enables this polymer to make complexation between the polar group and cations, forming a polymer electrolyte blend [12,13]. Several properties of the blended polymer electrolyte, for instance, chemical stability, quite large dielectric strength, satisfactory charge-storage capacity (CSC), dopant dependence of electric, abrasion resistance and optical characteristics, have drawn the attention of many researchers to focus on it [14]. Developing benign electrolytes is one of the goals that all researchers have taken into consideration in the field of secondary lithium batteries. Nowadays, among many other new classes of useful materials, SPEs are still one of the hottest topics that many research groups have focused on [15].

The modification of SPEs is addressed to be eligible for applications in lithium batteries and other electrochemical devices [16]. The commercialization of SPEs has faced two main barriers, which are low ionic conductivity and insufficient mechanical property [17].

**Figure 1:**
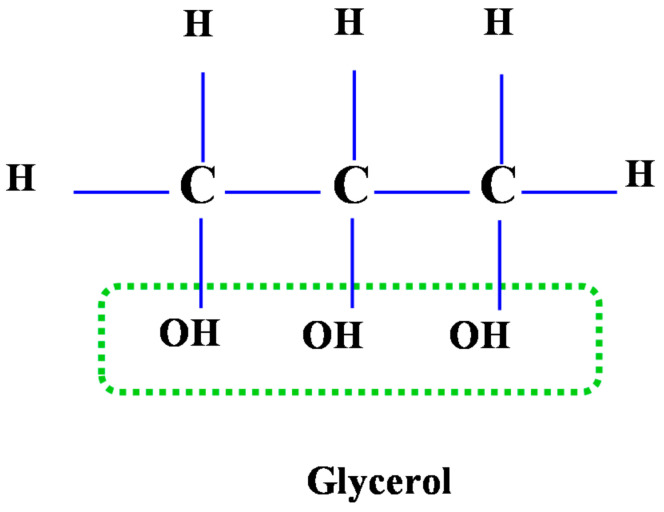
Structure of glycerol.

It has previously been emphasized that glycerol as a chemical plasticizer improves the ionic conductivity of polymer-based electrolytes and enhances salt dissociation [18]. The latter has resulted from columbic force weakening between cations and anions of the salt; thereby, ion concentration increases. Chai and Isa [19] confirmed the influence of glycerol addition on ionic conductivity improvement and the mechanical strength increasing of a particular electrolyte film. Figure 1 shows that glycerol contains multi-hydroxyl moiety (-OH) in the structure that facilitates free ion movement in the polymer electrolytes (PE). Mattos et al. [20] also documented the conductivity improvement of electrolyte systems in this range (10^−7^ S cm^−1^–10^−5^ S cm^−1^) due to adding glycerol.

An electrical double-layer capacitor (EDLC) is a supercapacitor device (SCDs), which is characterized by the simplicity of fabrication and it has been widely studied [21,22]. Moreover, the increasing demand for wearable electronics and flexible EDLC further encouraged research groups to modify various components of the device [23,24]. Among different components of the device, a particular attention is given to the electrolyte of the EDLC. Many studies have shown that polymer based electrolyte can be considered a suitable candidate for the fabrication of flexible EDLC with good performance under bending, twisting and folding conditions [24,25,26,27]. Regarding electrode fabrication to be utilized in EDLCs, various carbon allotropies with relatively high surface areas (porous materials) and enough electrical conductivity can be used. These materials with these characteristics can encompass a considerable quantity of charged ions via adsorption and desorption processes [28,29]. Several crucial properties, such as equivalent series resistance (*ESR*), specific capacitance (*C_s_*) and power (*P_d_*), and energy (*E_d_*) densities, must be taken into consideration in order to evaluate the efficiency and the performance of EDLCs. The two important terms *E_d_* and *P_d_* of energy devices generally refer to the amount of energy available and the energy delivery rate, respectively; which is usually expressed in the well-known Ragone plot [30,31]. EDLCs have extensively been applied to large-scale communication apparatuses, electronic technologies and hybrid automobiles [32,33]. Therefore, the efficiency and performance of EDLCs have to be modified via the usage of several materials such as activated carbon aerogels, graphene, carbon nanofibers and SPEs. These materials possess sufficient mechanical strength and electrochemical capacitance. Thus, the physiochemical properties of polymer electrolyte based EDLC should be carefully tuned and optimized in order to reach the industrial level, which is crucial from both practical and scientific viewpoints [34,35].

The most popular and usable carbon material is activated carbon (AC), a relatively perfect active material for electrode fabrication in EDLCs. This carbon material is characterized by a large surface area (usually 2500 m^2^/g), high conductivity and cost-efficiency [36,37]. Besides, carbon black (CB) is highly utilized as conducting material for fabricating the electrodes. It acts as a support filler. This material has a surface estimated to be in the range of 25 to 1500 m^2^/g, which is lower than AC [38].

The widespread use of electronic devices and all the related electronic wastes have resulted in climate change and negatively impacted on the environment. This urged the scientific community to devote large effort to overcome these issues and minimize livelihood implications and reduce the global worming effect using engineered biodegradable materials. This work is aiming at controlling various properties of biodegradable polymer electrolyte, so that an environmental friendly material for energy devices such as EDLC is viable. In this work, the eligibility of PVA:NaI:glycerol polymer electrolyte for EDLC device is addressed. Earlier studies highlighted that salts accompanying with low lattice energy should be considered for polymer electrolyte preparation. The selection of NaI (675 KJ/mole) over NaCl (796 KJ/mol), NaNO_3_ (755 KJ/mol), and NaOH (887 KJ/mol) is interrelated to the low lattice energy of NaI. Therefore, massive quantities of NaI can certainly be dissolved in host PVA polymer. Enormous amount of salt harvests additional charge carrier density, which is decisive to increase the DC conductivity value. Herein, the addition of a 50 wt.% of NaI salt has been applied to the PVA with different glycerol concentrations to obtain sodium ion-conducting plasticized electrolytes. Electrical impedance spectroscopy (EIS) was used to calculate ionic conductivity of PVA:NaI:glycerol-based polymer electrolyte at room temperature (RT). The dielectric properties of the prepared electrolytes were also intensively explained. The relatively high conducting electrolyte is employed in the EDLC assembly and tested its performance.

## 2. Experimental

### 2.1. Material and Preparation of SPE Plasticized Films

The main raw material used was PVA with an average molecular mass of 35,000 g/mol, purchased from Sigma Aldrich. Sodium iodide (NaI) salt was used to provide Na^+^ ions in the plasticized polymer electrolyte. In the preparation of plasticized polymer electrolytes, 1 g of PVA was putin 20 mL of distilled water and dissolved at 80 °C. Then, the mixture was left to reach the ambient temperature. A solution of 50 wt.% of NaI dopant was added to the polymer solution and stirred steadily until a clear solution was obtained. A series of 10 to 50 wt.% of glycerol plasticizer was then added separately to the PVA:NaI electrolyte. Ultimately, the solution mixtures were poured into a series of clean and dry clean glass Petri dishes to produce films. Afterward, the films were left to dry gradually at an ambient temperature to gain PVA:NaI:glycerol electrolyte films. Table 1 summarizes the weight ratio of samples.

### 2.2. Electrochemical Impedance Spectroscopy (EIS)

For the plasticized electrolytes’ impedance characterization, electrical impedance spectroscopy (EIS) was used in the evaluation using HIOKI 3532-50 LCR HiTESTER (50 Hz ≤ f ≤ 1000 kHz) at RT. The process of EIS measurements were comprised of putting the film between two stainless steel discs. From these measurements, both ionic conductivity and dielectric behaviors of the electrolytes were determined.

### 2.3. Transference Number Measurement (TNM)

For Transference Number Measurement (TNM) measurements, the polarization of stainless steel (SS) | conducting SPE | (SS) cell by fixing the working voltage at 0.80 V was implemented, and both ion (*t_ion_*) as well as electron (*t_el_*) transference numbers (TNM) were obtained. For these measurements, V&A Instrument DP3003 digital DC source was used at RT. The *t_ion_* can be determined from [12]:

(1) $$t_{ion}=\frac{I_{i}-I_{ss}}{I_{i}}$$

(2) $$t_{el}=1-t_{ion},$$

where the initial and steady-state currents are designated as *I_i_* and *I_ss_*, respectively.

### 2.4. Linear Sweep Voltammetry (LSV)

The Linear Sweep Voltammetry (LSV) is informative and used to establish the potential stability of the PE. Digi-IVY DY2300 potentiostat was employed, and the sweep voltage range was determined at a sweep rate of 50 mV/s.

### 2.5. Fabrication of EDLC

A homogeneous mixture of 81.25% activated carbon (AC) and 6.25% carbon black (CB) was obtained by grinding using a planetary ball miller. Meanwhile, a homogeneous solution of 12.5% of polyvinylidene fluoride (PVdF) and 15 mL of N-methyl pyrrolidone (NMP) was obtained by stirring. This solution was added to the prepared powder and then poured into the PVdF-NMP solution. A thick black solution mixture from continuous stirring was obtained. The resulting mixture was coated on an aluminum foil through a doctor blade and dried in an oven at 60 °C. The created electrodes were stored in a desiccators to remove moisture. The EDLC assembly consisted of two electrodes with a surface area (2.01 cm^2^) and the conducting electrolyte. In the CR2032 coil, the cell was packed in a Teflon case.

### 2.6. Characterization of the EDLC

The *CV* experiment was run at different scan rates from (10 to 100) mV/s to investigate the scan rate impact on the specific capacitance (*C_CV_*), and the equation used to calculate *C_CV_* is represented by [21,30,31]:

(3) $$C_{CV}=\int_{V_{i}}^{V_{f}}\frac{I\left(V\right)dV}{2mv\left(V_{f}-V_{i}\right)},$$

where *V_i_* and *V_f_* are 0 V and 0.9 V, respectively, and *I(V)dV* is the *CV* response area obtained for the integration function of Origin 9.0 software. The *v* and *m* are the scan rate and mass of active material, respectively. 0.5 mA/cm^2^ as a constant current density is applied to the EDLC. To obtain the decisive parameters of EDLC; specific capacitance from the *CV* and charge–discharge (*C_CD_*), *ESR*, *E_d,_* and *P_d_* are determined using these expressions shown below [21,30,31]:

(4) $$C_{CD}=\frac{i}{sm}$$

(5) $$ESR=\frac{V_{d}}{i_{}}$$

(6) $$E=\frac{C_{s}V^{2}}{2}$$

(7) $$P_{}=\frac{V^{2}}{4m(ESR)},$$

where *s* and *i* are a gradient of the discharge region and applied current, respectively; *V_d_* and *V* are the drop potential and applied voltage, respectively.

## 3. Results and Discussion

### 3.1. Impedance and Ion Transport Parameters Study

Insight into the charge transport mechanism in ion-conducting materials is of great importance fundamentally and technologically. For this purpose, electrochemical impedance spectroscopy (EIS) was used as one of the powerful techniques [39,40,41]. In this work, EIS was used to evaluate the electrical properties of the ion-conducting polymer membrane. This polymer membrane and others now have broad utilization in a range of solid–state electrochemical devices [42,43].

**Figure 2:**
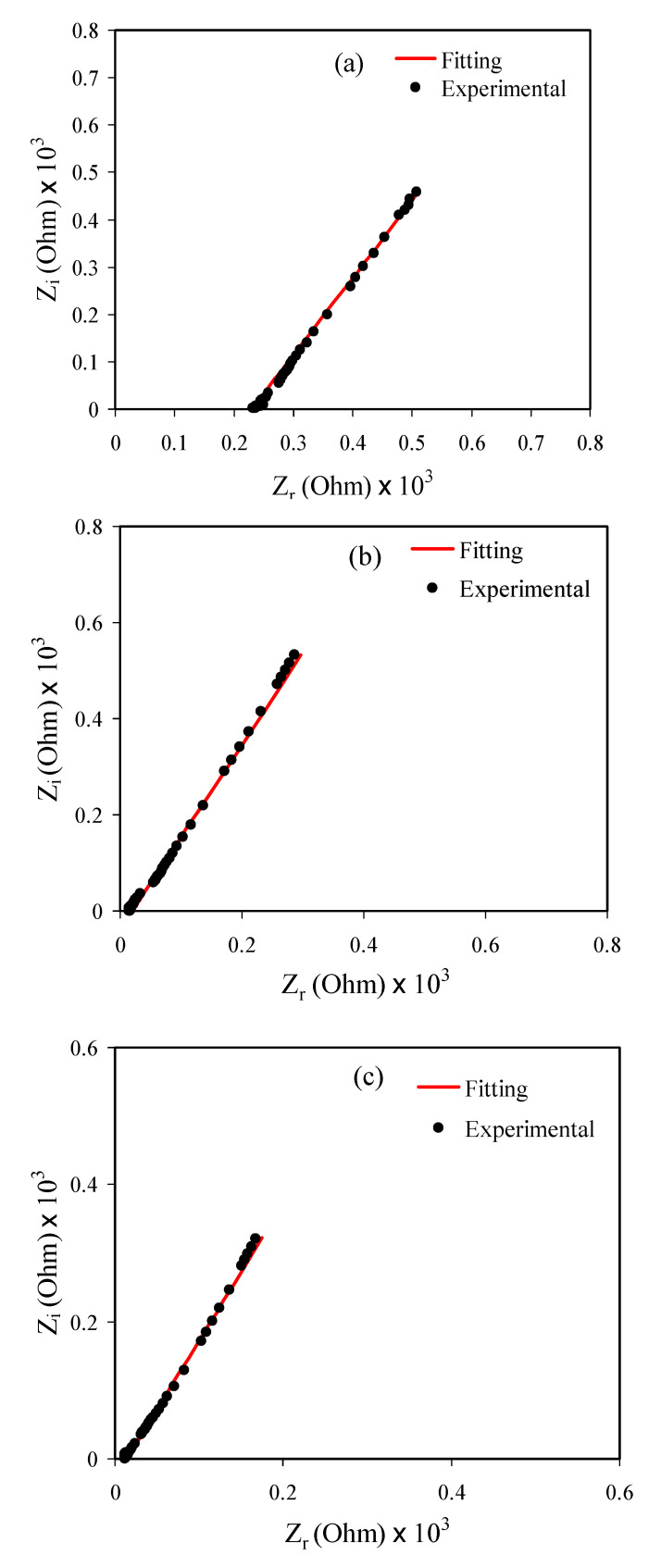

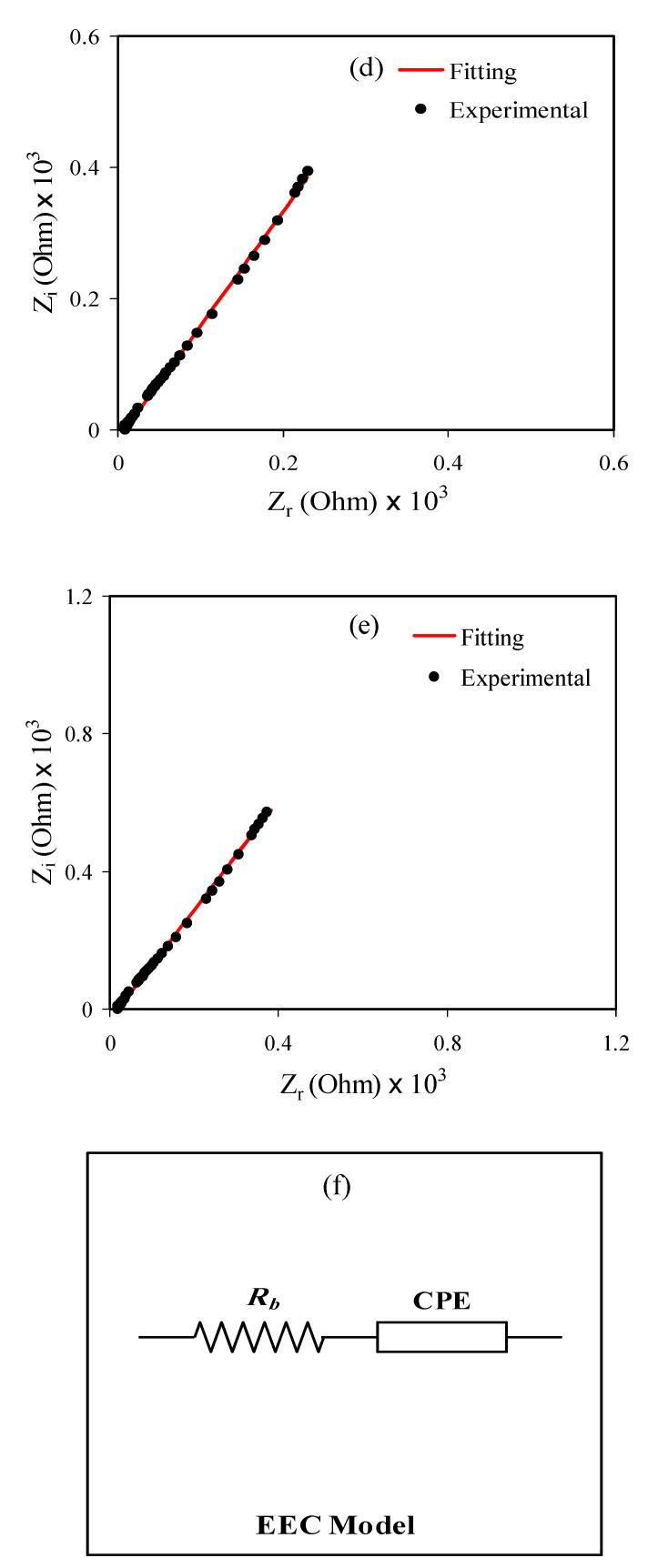
Impedance plot for the prepared polymer electrolyte systems (**a**) PVNA1 (**b**) PVNA2 (**c**) PVNA3 (**d**) PVNA4 (**e**) PVNA5 and (**f**) EEC model.

Figure 2a–e depicts the impedance spectra (i.e., Z_i_ as a function of Z_r_) for all samples. A linear line (or spike) at the low-frequency region appears due to the electrode blocking occurrence [44,45,46]. The pseudo-capacitance causes this phenomenon at the electrode/electrolyte interface regions. The *R_b_* values can be calculated from data analysis using the spike intercept with the spectra’s real axes. This response in the complex impedance plot is ascribed to the ionic conductivity whenever approaching the zero-phase angle [40]. Ultimately, the values of DC conductivity (σ_dc_) values can be determined using the equation below [30]:

(8) $$\sigma_{dc}=\left(\frac{1}{R_{b}}\right)\times \left(\frac{t}{A}\right),$$

where the surface area and thickness of the sample are symbolized by *A* and *t*, respectively. To decide on the eligibility of an electrolyte for utilization in electrochemical devices, the value of ionic conductivity has to be known as a critical factor. The plasticizer quantity is critical in enhancing the conductivity of the electrolyte (see Table 2). It is intuition that two factors govern the conductivity; charge carrier numbers and ion mobility (in other words, size, and electronegativity of ion).

In the current study, 50 wt.% of NaI salt is optimum to provide enough charge carrier density. It has been well documented that polymer electrolytes possess high conductivity in the presence of ions. The mathematical relations of conductivity in one side with charge number density and ion mobility on the other side formulated can be shown below [3]:

(9) $$\sigma =\sum_{i}n_{i}q_{i}\mu_{i},$$

where $n_{i}$, $q_{i}$, and $\mu_{i}$ represent the charge carrier number (or only charge number), electron charge, and the mobility of the ion (here *i* is the identity of the ion), respectively [5]. From Equation (9), it is evident that a direct proportionality among conductivity (σ) and charge number ($n_{i}$) and the ionic mobility ($\mu_{i}$) in electrolytes is present. The former study has emphasized that the high number of charges results from salt dissociation using volatile solvents in the matrices of appropriate polymers that are compatible [47]. The trend of decreasing *R_b_* is caused by increasing glycerol content ranging from 10 to 40 wt.%. This can be considered as an indication of glycerol’s effect in providing a high amount of free ions throughout the polymer bodies by softening the polymer backbone. The glycerol plasticizer can dissociate more salts and disrupt hydrogen bonding between polymer chains. Thus, this improves the overall amorphous phase of the prepared samples, which acts as a pathway for ion conduction. Additionally, more free ion will be available for conduction [18,19].

The electrical equivalent circuit (EEC) model was studied to have better insight into the ion migration. This model is simple and it can provide a clear picture of the whole polymer electrolyte system [41,48]. From the impedance plot of the samples (Figure 2e) the equivalent circuit (EC), which comprises $R_{b}$ for the charge species in the prepared polymer electrolyte systems and a constant phase elements (CPE) is series, as presented in Figure 2f. Figure 2a–e shows the fitting of the experimental data point of each film’s impedance spectra.

The impedance of CPE (*Z_CPE_*) can be imagined using the equations shown below [15,31,49]:

(10) $$Z_{CPE}=\frac{1}{C\omega^{p}}[\cos(\frac{\pi p}{2})-i\sin(\frac{\pi p}{2})]$$

where *C*, *ω*, and *p* are the CPE capacitance, the angular frequency, and the deviation of the spectra from the axis, respectively.

For the B3-labeled electrolyte sample, from the only spike, *R_b_* is combined in series with CPE, the mathematical impedance relationship can be expressed as follow [31]:

(11) $$Z_{r}=\frac{\cos(\frac{\pi p}{2})}{C\omega^{p}}+R_{b}$$

(12) $$Z_{i}=\frac{\sin(\frac{\pi p}{2})}{C\omega^{p}}$$

The above equations can also determine the fitting parameters (CPE1 and CPE2) and accurately measure the $R_{b}$ values. The determined CPE values for the **PVNA1**, **PVNA2**, **PVNA3**, **PVNA4**, and **PVNA5**-labelled electrolytes, respectively, are presented in Table 3. The relatively maximum conductivity value obtained for the **PVNA4** electrolyte sample indicates the electrolyte’s eligibility for ion-conducting devices. This is because the conductivity of polymer electrolyte plays the key role in the overall performance of the electrochemical devices [50]. A good conductivity range must lie between 10^−3^ to 10^−5^ S cm [50].

All transport parameters, such as number density (*n*), diffusion coefficient (*D*), and mobility (*μ*) of the ions are determined from the impedance data consisting only of a spike using equations shown below [51]:

The following relationships used to calculate the diffusion coefficient of the ion carriers [51]:

(13) $$D=D_{{}^{\circ}}\exp\{-0.0297{\left[\ln D_{{}^{\circ}}\right]}^{2}-1.4348\ln D_{{}^{\circ}}-14.504\}$$

and

(14) $$D_{{}^{\circ}}\;=\left(\frac{4k^{2}l^{2}}{R_{b}{}^{4}\omega_{\min}{}^{3}}\right).$$

Here, l is the film thickness of the electrolyte, and *ω*_min_ is the angular frequency referring to the minimum *Z_i_*.

The mobility (*μ*) of the ion carriers is determined from Equation (15) [51],

(15) $$\mu \;=\left(\frac{eD}{K_{b}T}\right),$$

where *T* and *k_b_* are the absolute temperature and the Boltzmann constant, respectively.

The DC conductivity of ions can be obtained from the following relationship [51]:

(16) $$\sigma_{Dc}=ne\mu .$$

Herein, it is easy to calculate the number density of ion carriers (*n*) using Equation (16).

Table 4 depicts the ion transport parameters and the corresponding *ω*_min_ values of the whole electrolyte systems.

Table 4 presents the diffusion coefficient *D* value where an increase was recorded with the addition of the glycerol content from 10 to 50 wt.%. A similar trend is obtained for *μ* as presented in Table 4. These rises in *D* and *μ* are strongly correlated to the chain flexibility enhancement with glycerol. The glycerol with high dielectric constant has the power to weaken the attraction force between ion species (cations and anions); in addition, it can disrupt bonding between the host polymer chains which enhance the segmental motion. These two factors will result in improving the overall conductivity of the polymer electrolyte upon the addition of glycerol. It is also noted that the *n* values increase as a result of conductivity enhancement. As documented previously, the higher the glycerol addition, the more tendency of the salts to dissociate to free ions; thereby, the number density of charge carriers increases as well [51]. Hence, a higher number of NH₄⁺ (*n_i_*) can be generated by NaI to the polymer. The rising dielectric constant of polymer electrolytes moving towards a low frequency at which the capacitance would increase was the important note reported [52]. The capacitance also increases with increasing the glycerol content. As the number density of free ions increases (see Table 4), the dielectric constant value increases, due to the high value of dielectric constant of the used glycerol, which can dissociate more salt and high carrier density can be achieved.

### 3.2. SPE Film Characteristic for EDLC Application

#### 3.2.1. Transference Number Measurement

**Figure 3:**
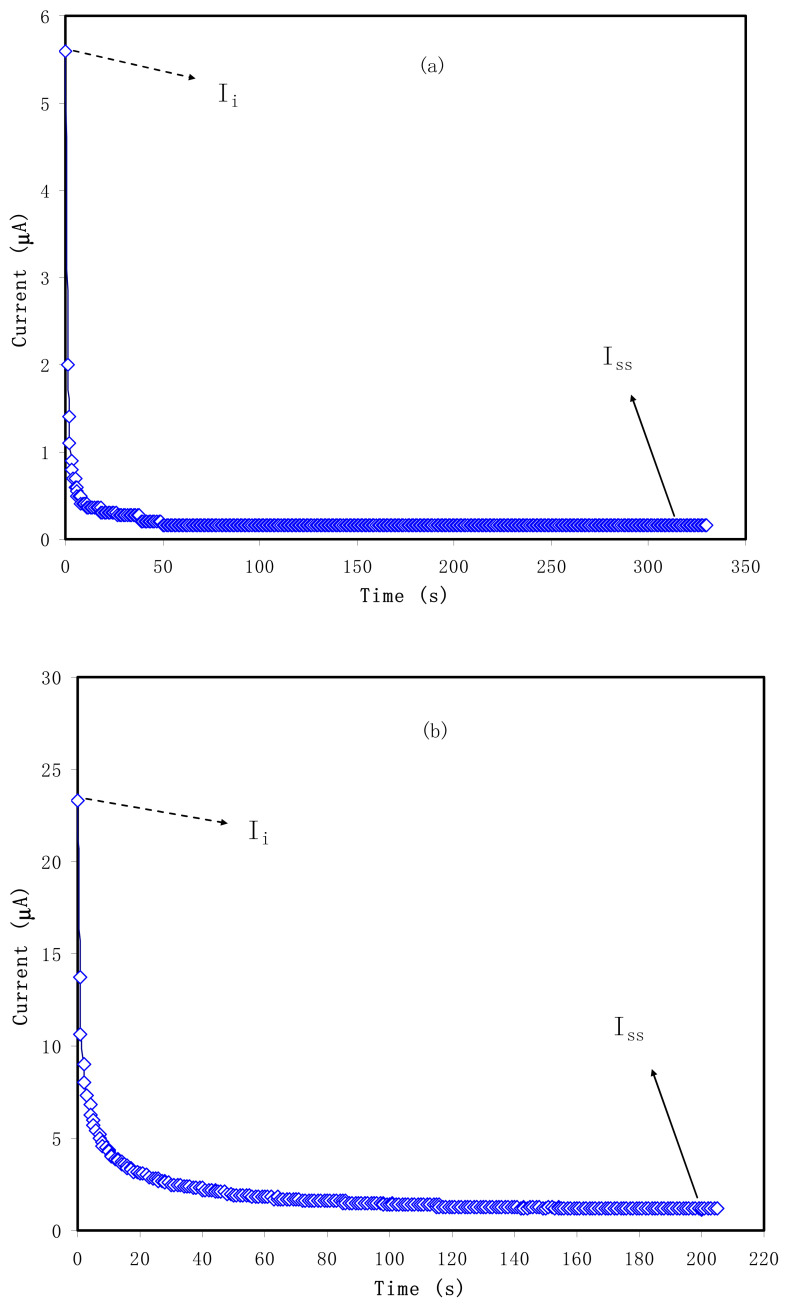
Current polarization plot for (**a**) **PVNA4** and (**b**) **PVNA5**.

The quantity of *t_i_* has to approach unity to utilize the electrolyte in EDLCs. In such systems, ions are the primary charge carriers compared to the electron. In principle, electron and ion are two types of charge-carrying species. Solid-state ionic is one branch of physics that deals with ionic transport properties of solids that possess reasonably high ionic conductivity [53]. Solid electrolytes (or super ion conductors, SICs) represent those materials characterized by the superiority of the ionic conductivity over electronic ones by recording 10^−6^ S cm^−1^ and 10^−12^ S cm^−1^, respectively, at room temperature [54,55]. Na^+^ and I^−^ ions are the two main species that perform adsorption at the AC electrodes’ surface in the present systems. In Figure 3a,b, the maximum current is recorded at the beginning and then a great fall occurs. Due to omitting the ionic conduction in the stainless steel electrodes, the decrease of the whole current in the transient profile is expected. The *t_i_* observed in our study has acceptable value and consistent with what has been reported in the literature for various polymer electrolyte systems [11,18,30,31]. The relatively high value of *t_i_* of the present electrolyte indicates to a large extent the eligibility of the electrolyte to be commercialized by using in EDLCs. In **PVNA4** sample *t_ion_* = 0.982, which is larger than *t_ion_* = 0.948 for **PVNA5** sample. Thus, **PVNA4** polymer electrolyte was used in the EDLC fabrication.

The *t_i_* value for the system composed of CS:MgCl_2_:glycerol was found to be 0.971. That is likely because of the dominancy of the charge transport in this polymer electrolyte by ions. Polu and Kumar [56] have given a *t_i_* of 0.96 for the system involving Mg(CH_3_COO)_2_ salt with PVA host. From the literature, it was recorded that I-carrageenan:Mg(NO_3_)_2_ [57] and PEG:Mg(CH_3_COO)_2_:CeO_2_ [58] possessed the *t_i_* value of 0.97. Thus the *t_ion_* accomplished in the existing study is of great prominence.

#### 3.2.2. LSV Analysis

**Figure 4:**
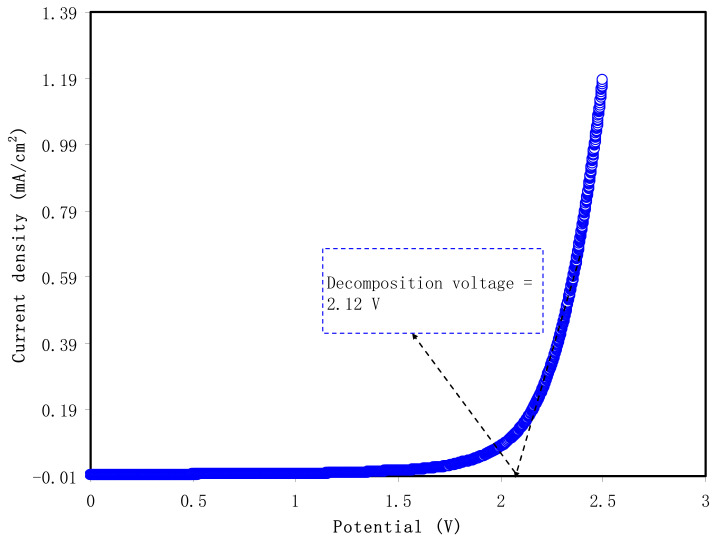
Linear sweep voltammetry (LSV) pattern and decomposition voltage for the highest conduction sample.

The voltage window as a measure of the polymer electrolyte system’s electrochemical stability is highly significant from a commercialization perspective [59,60]. Figure 4 shows LSV response as potential dynamic scanning in a cell containing PVA:NaI:GL electrolyte. The LSV pattern of the tested sample was providing a good insight into the suitability of used sample for the electrochemical device applications, particularly EDLC. This is because during the charge–discharge process of the EDLC, a high voltage will build up on the electrolyte, which might result in the decomposition of the used electrolyte and cause device failure. From Figure 4, one can note that there is no considerable increment in the value of current up to 2 V. However, there is a sharp rise in the value of current when the applied voltage reaches above 2.12 V. This exponential ascent in current is the clear sign of the decomposition of the electrolyte within the electrodes. This means that the range of voltage window for polymer blend electrolytes is around 2.12 V, which is enough for utilization in EDLCs [61]. Monisha et al. [62] have defined the threshold voltage, that is, voltage window, as the range of electrochemical stability within which current flow through the cells leads not to electrolytic decomposition. In other words, the solvent evaporation, leakage of liquid, and relatively low electrolyte breakdown voltage (<1 V) of electrolytes, in particular, are detrimental, causing low SCDs performance [63].

### 3.3. EDLC Characteristics

#### 3.3.1. Cyclic Voltammetry (CV)

**Figure 5:**
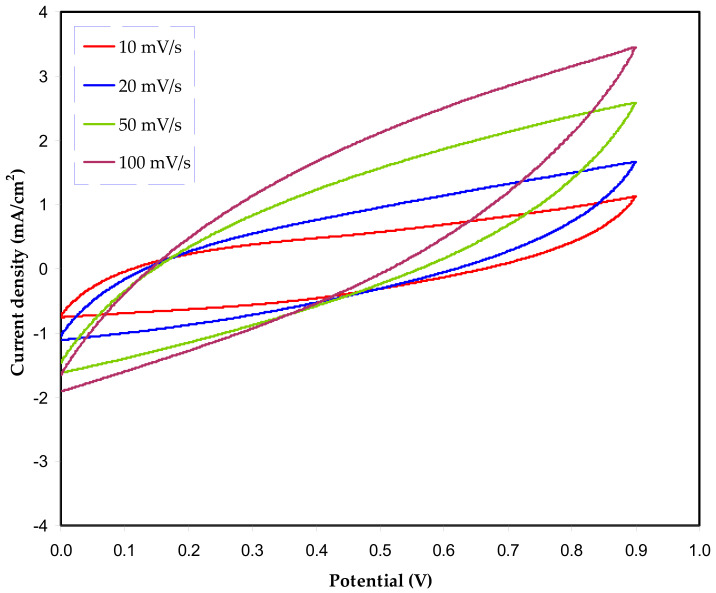
Leaf shape cyclic voltammetry plot of the fabricated electrical double-layer capacitor (EDLC) system based on the highest conducting polymer electrolyte.

To evaluate the performance of the EDLC, CV was recorded. Figure 5 shows the influence of scan rate of the main feature of CV shape. It is seen that the main feature of the CV of leaf shapes has remained similar at all chosen scan rates. A perfect capacitor is characterized by a common feature that is an ideal rectangular shape of the CV electrochemical signal. Nevertheless, in reality, the main features of CV have changed dramatically due to the nature of electrode surface (porosity), which causes changes in internal resistance. This alteration in CV response results in changing EDLC performance as well as efficiency. Besides, the I-V response is also impacted [64].

It can be noticed that no redox peak is observed over the voltage range of scanning. This indicates the occurrence of charge storing via anon-faradaic procedure, which is a basis of EDLCs. According to this procedure, both cations and anions adsorb and desorb at electrode surfaces, that is, intercalation and deintercalation processes are absent [65].

Table 5 presents the *C_cyc_* values, where the maximum is recorded at a low scan rate and gradually decreasing as the scan rate increases. In principle, at a low scan rate, a stable double-layer charge forms from the adsorption of ions at the interfacial region. Here, the common question arises why an almost perfect plateau is obtainable at a low scan rate. The answer is the building up of a thick diffuse layer from ion adsorption at the interfacial region. This also causes small ohmic resistance. On the contrary, a thin, diffuse layer at a high scan rate is formed and consequently small capacitance is resulted [66].

#### 3.3.2. Charge-Discharge of the EDLC and Other Parameters

**Figure 6:**
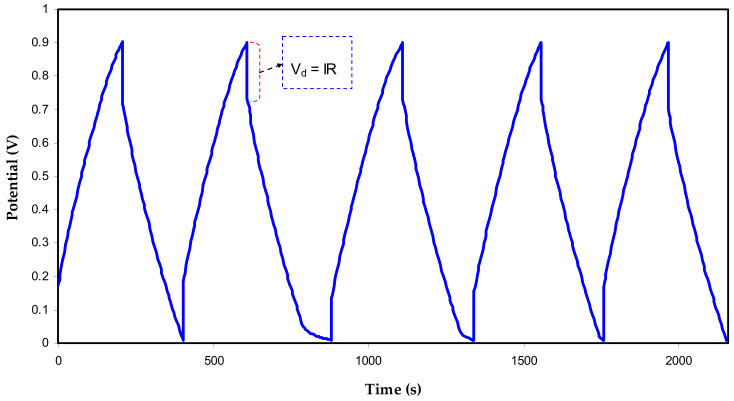
Charge-discharge profile of the designed EDLC at specific cycles.

The charge-discharge plot’s response at a range of cycles of the EDLC is depicted in Figure 6. Charging and discharging of EDLC was occurred in this range (0–0.9 V), respectively, at a constant current density, that is, 0.75 mA/cm^2^. The curve’s main feature is linearity from which the slope can be determined, and capacitor behavior can be evidenced for the mechanism of storage in EDLC [67]. More observation comprises a drop in voltage (*V_d_* = *IR*) before starting the discharge process and producing an ohmic drop. Another desired property of EDLC assembly is the low equivalent series resistance (*ESR*). This type of resistance originates from several reasons; bulk resistance of the electrolyte and a drop in *IR* between the electrolyte and current collectors [68,69].

**Figure 7:**
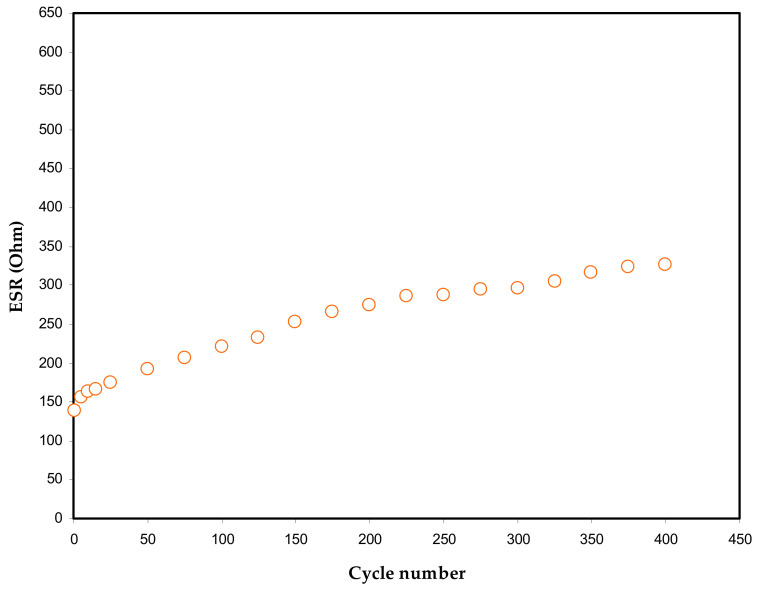
Equivalent series resistance trend of the fabricated EDLC over 400 cycles.

More insight into the EDLC assembly is obtained from the *ESR* response vs. cycle numbers Figure 7. At the 1st cycle, the *ESR* value of 91.2 Ohm is recorded and then remains unchanged until the 50th cycle with the average value of 95.4 Ohm for the EDLC assembly. The *ESR* values rise as the cycle number increases until the 100th cycle, reaching 101.7 Ohm and becomes constant at the 400th cycle. During the experimental cycling course, a minute increase in ESR occurs throughout the 400th cycle; similarly, the same trend is recorded for the specific capacitance. It is interesting to note that small record data of *ESR* suggests a satisfactory EDLC assembly with compatible electrolyte|electrode contact. This means that the overall mechanism is the migration of ions from the bulk electrolyte region to the activated carbon surface. Building up the space charge double-layer had a relatively low internal resistance [70].

**Figure 8:**
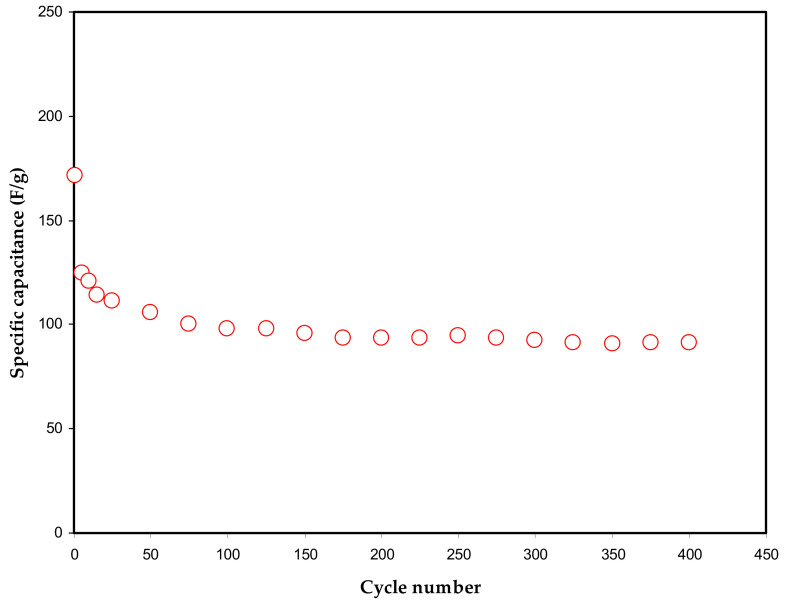
Variation in specific capacitance throughout 400 cycles.

The capacitance is expressed by the ratio of change in the electric charge corresponding to its electric potential in a given system. The *C_cd_* value of the EDLC assembly can be acquired from the response, as shown in Figure 8. As expected, the capacitance value is 169 F/g at the 1st cycle. This value is relatively large compared to that recorded for other PVA-based electrolytes incorporated with ammonium salts [21,37,50]. After the 1st cycle an enormous lessening of *C_spe_* was detected. A former study recognized that ion aggregate formation or ion pairs could be accountable for the deterioration of electrical conductivity [71]. It was proven that ion pairs could block the ion transportation through the polymer electrolyte, therefore impacting the degree at which ions are adsorbed at the carbon pores. Accordingly, this drops the growth of ion adsorption at the electrodes-electrolyte boundaries and thus reduces the *C_spe_* value [1,72].

Notably, an average value of 97.2 F/g is recorded as a constant value of *C_cd_* during the whole 400 cycles. This relatively high value of *C_cd_* can be attributed to the large surface area of activated carbon (2500 m^2^/g). More explanation for this situation comes from the fact that many free ions from NaI salt enable the electrode surface to occupy the electrode surface via an adsorption/desorption mechanism.

Francis et al. [73] have recently studied PVA-Mg(CF_3_SO_3_)_2_ in an EDLC assembly with recording a *C_cd_* value that lies in the range 15 to 45 F/g. Pandey et al. [59] examined magnesium salt-based EDLC assembly that gave a *C_cd_* value in the range of 31 to 41 F/g. Another studied system consisted of a cellulose derivative as the polymer host (hydroxylethyl cellulose, HEC) and Mg(CF_3_SO_3_)_2_ as the ionic provider and silica nanoparticles as additives that recorded a *C_cd_* of 25.1 F/g [66].

**Figure 9:**
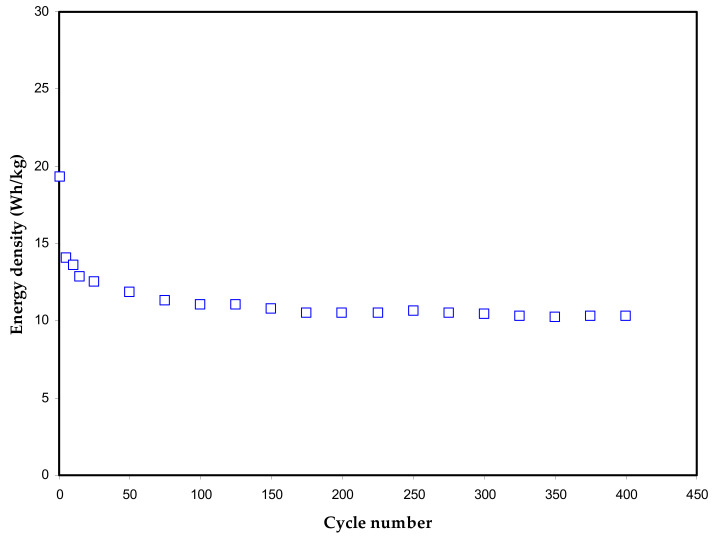
Energy density plot of the fabricated EDLC over 400 cycles.

The energy density (*E*) means measuring the speed up of energy up-taking in a confined specific space per unit volume. Figure 9 presents *E* variations over a wide range of 400 cycles. The energy density is directly proportional to the specific capacitance, as mathematically shown in Equation (6). This is the cause of *E* and *C_cd_* similar trends, as shown in Figure 9.

An EDLC assembly containing polyacrylonitrile-magnesium chloride (MgCl_2_) electrolyte with the documented energy density of 5 Wh/kg was examined by Bandaranayake et al. [74]. Winie et al. [75] reported chitosan-based EDLC assembly possessing an energy density in-between (0.57–2.8 Wh/kg) that corresponds to current densities of 2 to 0.6 mA/cm^2^, respectively. The literature recommends that reaching an energy density of (5.48 Wh/kg) is desired. Another important notice is that the values of *E* and *C_cd_* responses are stable, evidencing least ion recombination to create ion pairs or agglomerates, which in turn minimize EDLC performance.

**Figure 10:**
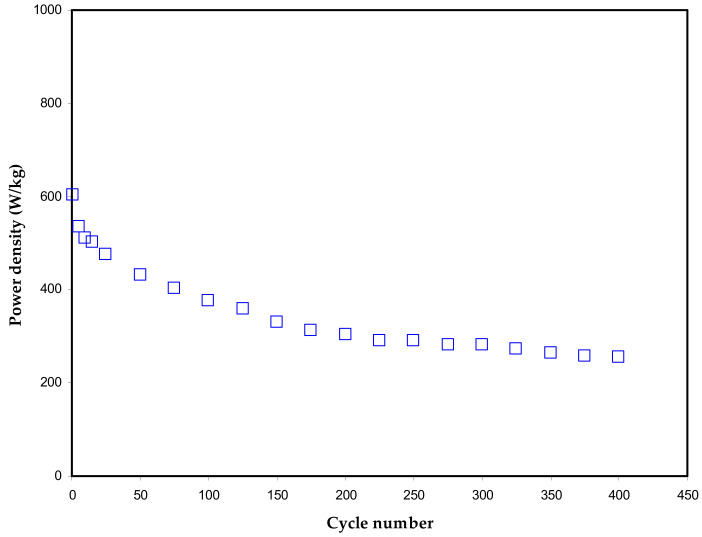
Power density trend of the fabricated EDLC over 400 cycles.

To fully evaluate the EDLC, the power density (P) has to be determined. It is practically essential that batteries are inferior to EDLCs based on low power density. This is because of the dominancy of ion adsorption over intercalation at the electrode surface in the EDLC. The trend of the power density of the EDLC assembly is exhibited in Figure 10. At the 1st cycle, the recorded value of power density (P) is almost 600 W/kg, whereas it gradually drops to 400 W/kg and becoming constant at the high cycle numbers. Yassine et al. [76] have established a strong relationship between ESR and power density by verifying the consistent trends of increasing the two parameters, as exhibited in Figure 10. More clearly, the high ESR value means facilitating energy delivery, confirming a large power density.

## 4. Conclusions

In conclusion, plasticized sodium ion-conducting polymer electrolytes based on the PVA via solution cast technique were fabricated successfully for energy storage application with enhanced *C_spe_* (169 F/g), energy density (19 Wh/kg) and power density (600 W/kg). The influence of plasticization on conductivity behavior was studied via the EIS technique. The conductivity of the plasticized films was determined via EIS experimental data fitted with the EEC model. The ion transport parameters at various concentrations of plasticizer achieved from EIS data establish plasticizer’s influence on transport properties. The sample with 40 wt.% of glycerol has shown the highest conductivity of 1.17 × 10^−3^ S cm^−1^. The TNM study revealed that ion transference number improved up to 40 wt.% of plasticizer with the highest *t_ion_* = 0.982 and then drops for the higher concentration of plasticizer. The LSV analysis revealed that the electrolyte is stable up to 2.12 V for device fabrication, and the sample represented good stability in a wide range of potential windows. The average value of specific capacitance for the assembled EDLC was found to be 97.2 F/g over 400 cycles. The charge-discharging response on the assembly EDLC device provides information on ion accumulation between the electrode and electrolyte. The absence of redox peaks was proved via CV, indicating the charge storing process mechanism that comprised ion accumulation at the interfacial region. The designed EDLC performed good energy and power densities with average values above 10 Wh/kg and 300 W/kg up to 400 cycles, respectively.

## Figures and Tables

**Table 1:** Sample designation with the salt plasticizer weight ratio.

Designation	NaI, wt.%	Glycerol Content, wt.%
PVNA1	50	10
PVNA2	50	20
PVNA3	50	30
PVNA4	50	40
PVNA5	50	50

**Table 2:** Determined ionic conductivity of the prepared samples.

Designation	Conductivity (S cm^−1^)
PVNA1	2.86 × 10^−5^
PVNA2	5.75 × 10^−4^
PVNA3	9.35 × 10^−4^
PVNA4	1.17 × 10^−3^
PVNA5	9.95 × 10^−4^

**Table 3:** The capacitance of *K* value of the fabricated polymer electrolyte systems.

Sample	*K* (*F^−^*^1^)	*C* (*F*)
PVNA1	3.28 × 10^4^	3.05 × 10^−5^
PVNA2	3.24 × 10^4^	3.09 × 10^−5^
PVNA3	2.08 × 10^4^	4.81 × 10^−5^
PVNA4	2.07 × 10^4^	4.83 × 10^−5^
PVNA5	2.75 × 10^4^	3.64 × 10^−5^

**Table 4:** Variety ion transport parameters of the prepared samples.

Sample	D (cm^2^ s^−1^)	μ (cm^2^ V^−1^ s)	n (cm^−3^)
PVNA1	3.03 × 10^−8^	1.18 × 10^−6^	1.52 × 10^20^
PVNA2	5.62 × 10^−7^	2.19 × 10^−5^	1.64 × 10^20^
PVNA3	6.60 × 10^−7^	2.57 × 10^−5^	2.27 × 10^20^
PVNA4	7.82 × 10^−7^	3.05 × 10^−5^	3.51 × 10^20^
PVNA5	6.06 × 10^−7^	2.36 × 10^−5^	2.63 × 10^20^

**Table 5:** Variation in capacitance value concerning scan rate for the fabricated EDLC device.

Scan Rate	Capacitance
0.1	10.425
0.05	17.649
0.02	32.464
0.01	46.867

## Data Availability

The data presented in this study are available on request from the corresponding author.
